# Supplementary material for: Single-step genome-wide association study for susceptibility to Teratosphaeria nubilosa and precocity of vegetative phase change in Eucalyptus globulus
Source: Front Plant Sci. 2023 Jul 3;14:1124768. doi: 10.3389/fpls.2023.1124768 (PMC10350686; doi:10.3389/fpls.2023.1124768)
Supplement: Supplementary file 3 [file DataSheet_1.pdf]

Supplementary Material

Supplementary Material of **"Single Step genome-wide association study for susceptibility to *Teratosphaeria nubilosa* and precocity of vegetative phase change in *Eucalyptus globulus*"**.

Marianella Quezada, Facundo Giorello, Cecilia Da Silva, Ignacio Aguilar and Gustavo Balmelli.

SUPPLEMENTARY FIGURES

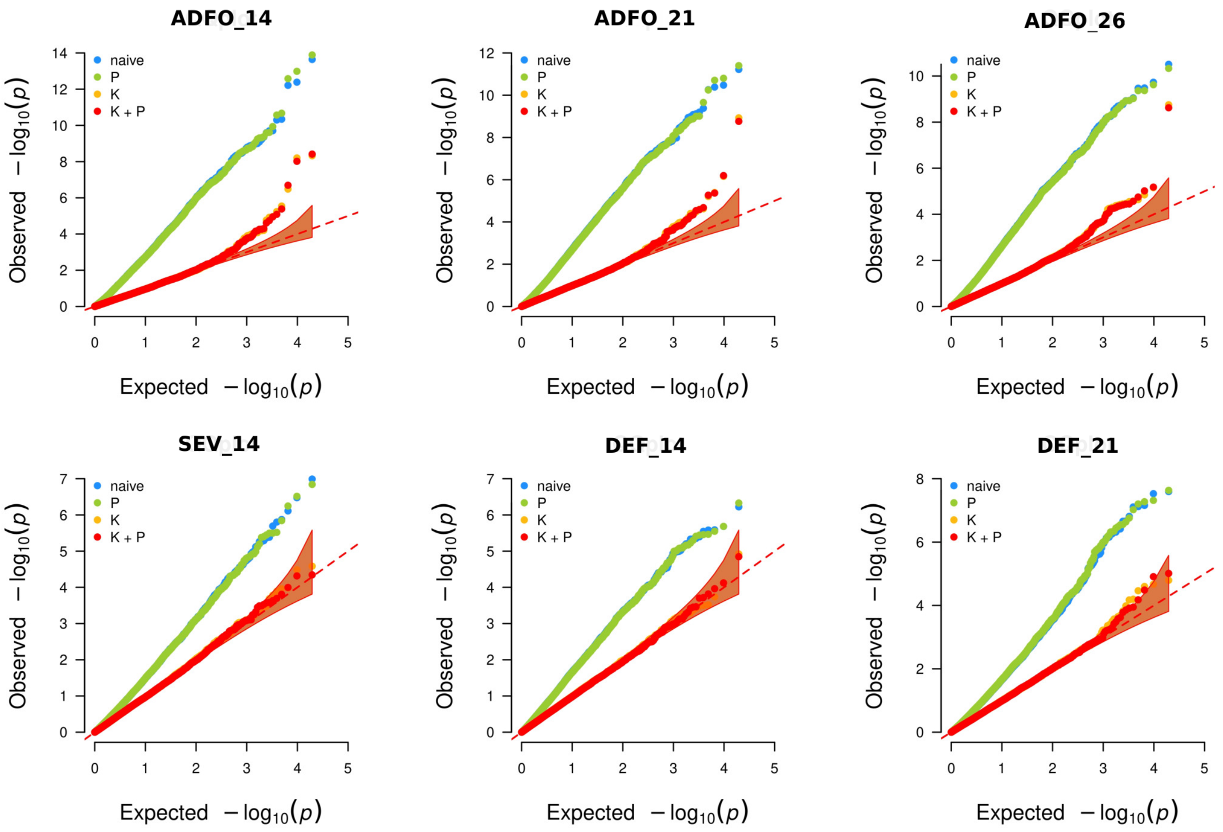

Figure S1: Quantile-quantile plots (QQ-plot) for Single-SNP GWAS models for six trait-age combinations. The trait codes for leaf spot severity (SEV), defoliation (DEF) and proportion of adult foliage (ADFO) are followed by the age of measurement in months. The observed association significance is plotted against the expected association significance for each SNP. The dashed red line represents the expected null distribution assuming no associations.

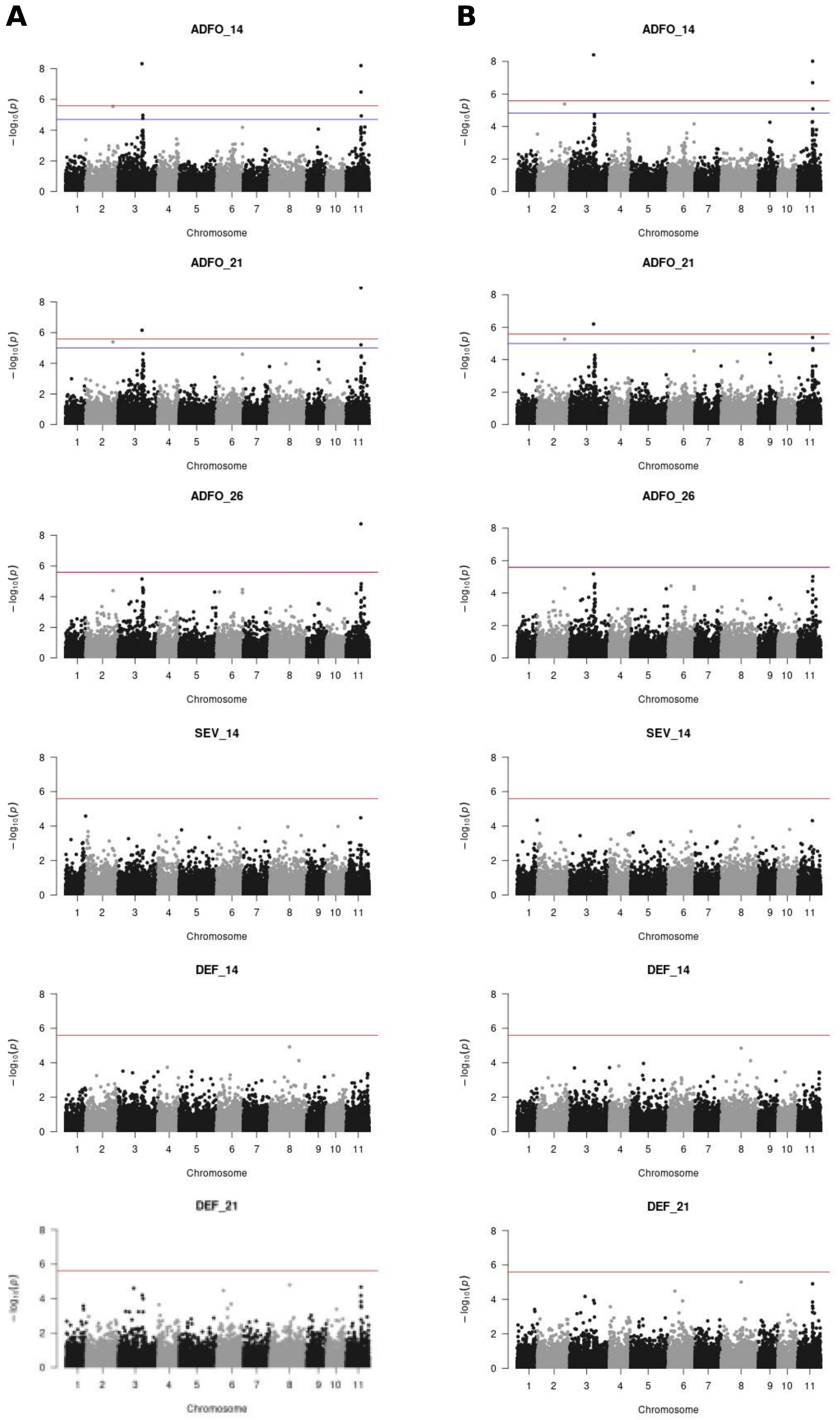

Figure S2: Manhattan plots for Single-SNP models of six trait-age combinations. (**A**) Single-SNP model adjusted for kinship matrix (model K) and (**B**) Single-SNP model adjusted for kinship matrix and population structure (model K + P). The trait codes for leaf spot severity (SEV), defoliation (DEF) and proportion of adult foliage (ADFO) are followed by the age of measurement in months. The x-axis represents SNP positions on the 11 *Eucalyptus gradis* chromosomes and the y-axis -log10 (p-values) from genotypic associations. The red horizontal line indicates the Bonferroni threshold (alpha = 0.05) and the blue horizontal line indicates a false discovery rate (FDR) at 5% threshold.

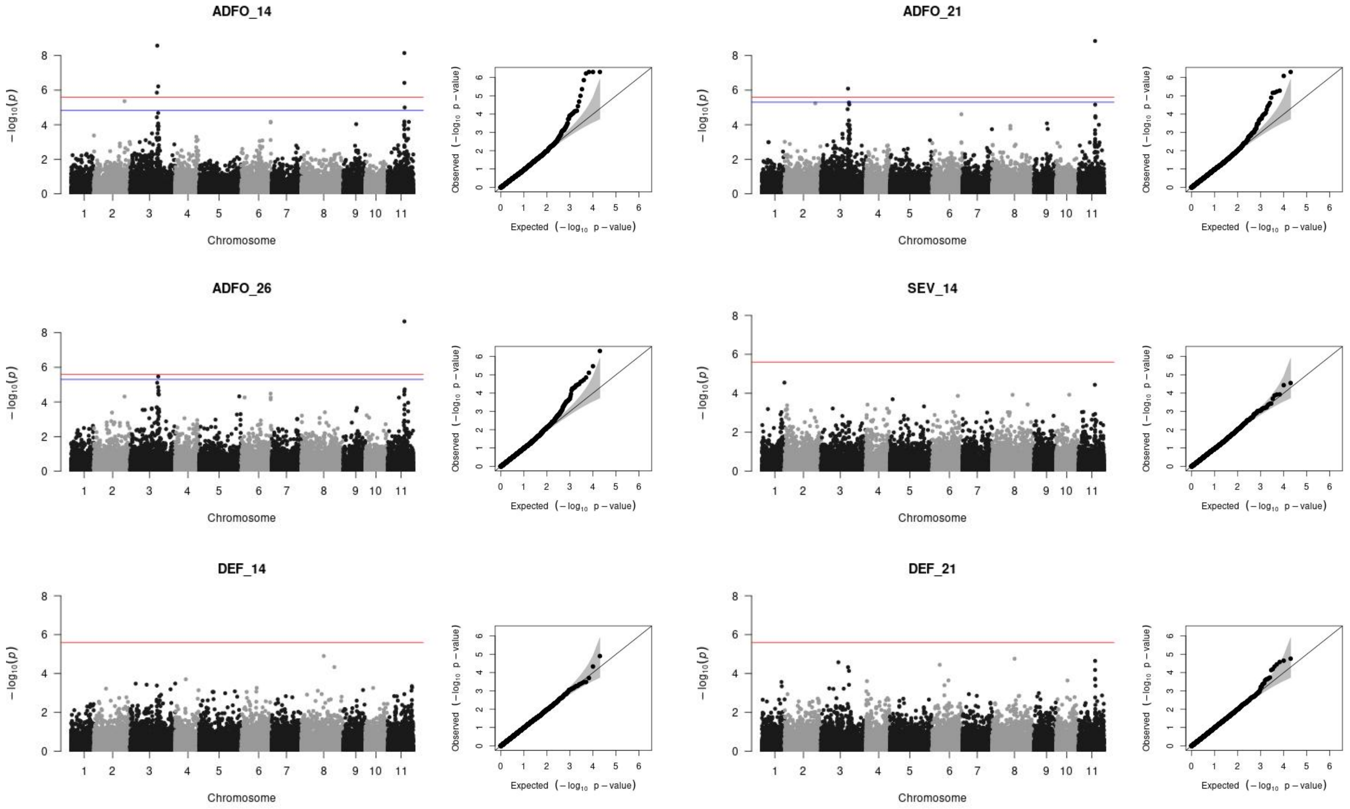

Figure S3: Manhattan plots and QQ-plots for GBLUP-GWAS in six trait-age combinations. The trait codes for leaf spot severity (SEV), defoliation (DEF) and proportion of adult foliage (ADFO) are followed by the age of measurement in months. The x-axis represents SNP positions on the 11 *Eucalyptus gradis* chromosomes and the y-axis -log10 (p-values) from genotypic associations. The red horizontal line indicates the Bonferroni threshold (alpha = 0.05) and the blue horizontal line indicates a false discovery rate (FDR) at 5% threshold.

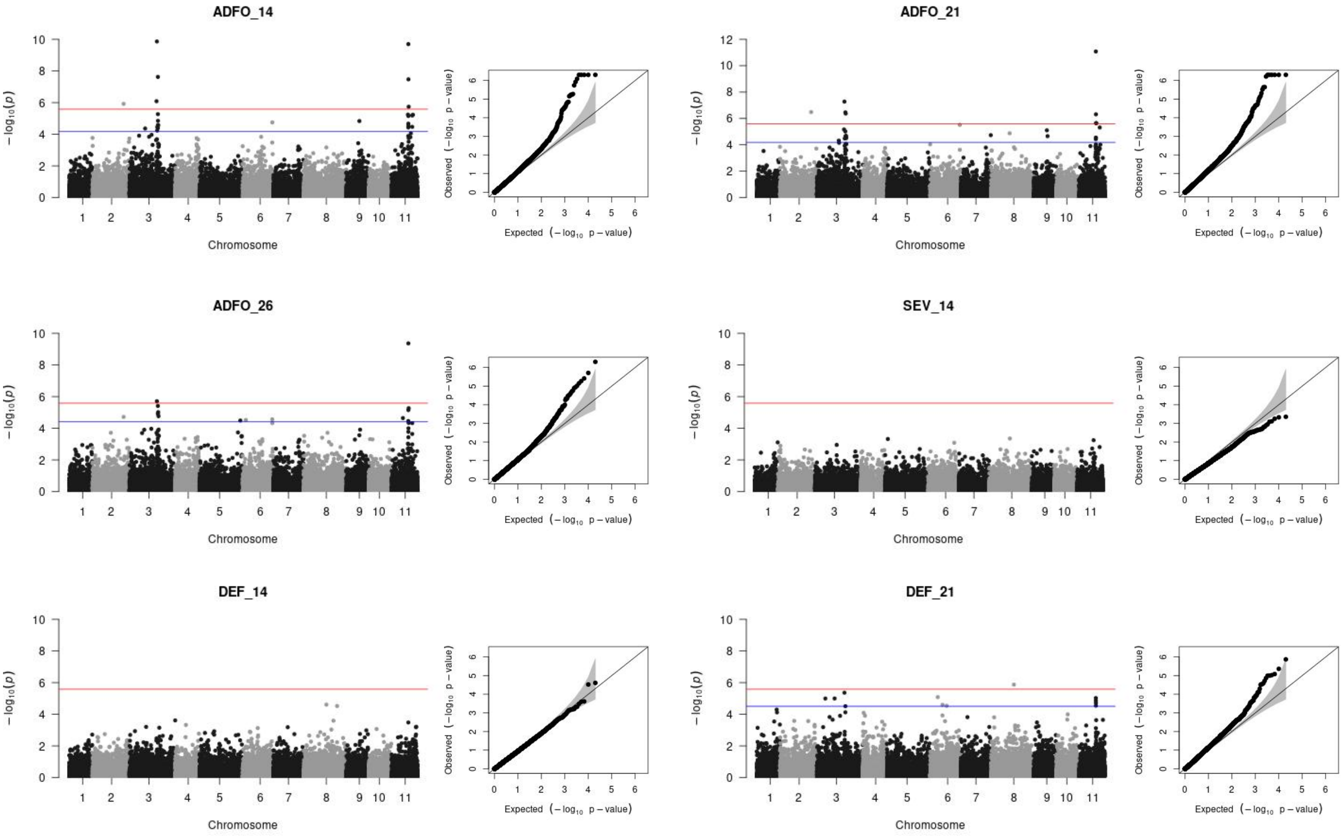

Figure S4: Manhattan plots and QQ-plots for single-step GBLUP association model (ssGWAS) in six trait-age combinations. The trait codes for leaf spot severity (SEV), defoliation (DEF) and proportion of adult foliage (ADFO) are followed by the age of measurement in months. The x-axis represents SNP positions on the 11 *Eucalyptus gradis* chromosomes and the y-axis -log10 (p-values) from genotypic associations. The red horizontal line indicates the Bonferroni threshold (alpha = 0.05) and the blue horizontal line indicates a false discovery rate (FDR) at 5% threshold.
